# Why are there so many explanations for primate brain evolution?

**DOI:** 10.1098/rstb.2016.0244

**Published:** 2017-07-03

**Authors:** R. I. M. Dunbar, Susanne Shultz

**Affiliations:** 1 Department of Experimental Psychology, University of Oxford, South Parks Road, Oxford OX1 3UD, UK; 2 Department of Computer Sciences, Aalto University, Espoo, Finland; 3 School of Biological Sciences, University of Manchester, Manchester M13 9PL, UK

**Keywords:** social complexity, foraging innovations, energetics, coalitions, multilevel sociality, social brain hypothesis

## Abstract

The question as to why primates have evolved unusually large brains has received much attention, with many alternative proposals all supported by evidence. We review the main hypotheses, the assumptions they make and the evidence for and against them. Taking as our starting point the fact that every hypothesis has sound empirical evidence to support it, we argue that the hypotheses are best interpreted in terms of a framework of evolutionary causes (selection factors), consequences (evolutionary windows of opportunity) and constraints (usually physiological limitations requiring resolution if large brains are to evolve). Explanations for brain evolution in birds and mammals generally, and primates in particular, have to be seen against the backdrop of the challenges involved with the evolution of coordinated, cohesive, bonded social groups that require novel social behaviours for their resolution, together with the specialized cognition and neural substrates that underpin this. A crucial, but frequently overlooked, issue is that fact that the evolution of large brains required energetic, physiological and time budget constraints to be overcome. In some cases, this was reflected in the evolution of ‘smart foraging’ and technical intelligence, but in many cases required the evolution of behavioural competences (such as coalition formation) that required novel cognitive skills. These may all have been supported by a domain-general form of cognition that can be used in many different contexts.

This article is part of the themed issue ‘Physiological determinants of social behaviour in animals’.

## 1. Introduction

Primate evolution has been dominated, as much as anything, by unusually large brains [1]. Over the past four decades, many explanations for the evolution of large brains have been proposed. Broadly, these explanations divide into four major themes, each with many sub-hypotheses of their own: genetic explanations (primates have large brains because a particular gene mutation allows them to grow large brains), developmental explanations (primates have large brains because their extended periods of parental investment allow them to grow large brains), ecological explanations (primates evolved large brains in order to cope with demanding environmental conditions), and social explanations (there is something intrinsically complex about primate sociality that requires a large brain). In many respects, the main problem associated with understanding why large brains have evolved has been the fact that there is an embarrassment of riches: there is empirical evidence to support every hypothesis. However, the fact that evidence can be adduced in favour of conceptually very different, mutually incompatible, explanations should alert us to the fact that something is amiss. Either there are confounding statistical issues such that we struggle to tease apart the causal relationships between a suite of highly correlated traits or there are conceptual issues stemming from the construction of alternative explanations.

Here we argue that the efforts to identify the correct explanations for brain size evolution have foundered on four major issues. The *first*, and undoubtedly most pervasive, has been a failure to distinguish between different levels of explanation (*sensu* [2]): authors often implicitly suppose that evidence for one hypothesis undermines all the other explanations even though the hypotheses under test may in fact be complementary and equally necessary. A *second* problem has been that too many studies still only provide evidence for a single hypothesis, and fail to test adequately between alternatives (see also [3]). A *third* issue has been a failure to specify exactly how brain size impacts on cognition (e.g. exactly what aspects of primate behaviour are *so* cognitively demanding) and how this relates to the underlying neurobiology [3,4]. The *fourth* issue has been a tendency to favour hypotheses that only apply to a subset of species, even within the primates. Explanations that apply only to special cases may be true, but they cannot be general explanations.

To highlight how these issues have obstructed our understanding of primate brain evolution, we evaluate each of the major explanations and specify exactly what assumptions and potential limitations underlie each in turn. In doing so, we develop a framework that articulates the various explanations within a single explanatory model. We shall argue that an important fulcrum in this is the energetic costs of evolving and maintaining both large brains and large groups: these are invariably ignored.

The test we must apply to any prospective explanation is that it can explain six key empirical findings: (i) that primates have larger brains relative to their body size than all other animals [1]; (ii) that some primates have larger brains than other primates [1]; (iii) that there is a remarkably robust quantitative relationship between brain (and especially neocortex) size and group size in primates (but not other mammals or birds) [5,6]; (iv) that primates have a peculiar form of bonded sociality that seems to be very different to that of other mammals [7–12], reflected in the fact that primate societies are highly structured in network terms (whereas those of most other mammals and birds are not) [13–16]; (v) that pairbonded monogamy in birds and mammals is associated with larger than average brain size for their orders [7,8]; and (vi) that some (but not all) species of primates exhibit novel technical competences [17,18].

Although many analyses do so, we should not ignore the relationship between group size and brain size in primates, because living in groups is extremely costly to animals. This is so for three reasons. First, increasing group size unavoidably increases competition and induces costs in terms of time required for foraging, travel and, in primates, social bonding, that place significant stress on the animals' ability to survive in a given habitat [19]. Second, an organism is an integrated biological system and any change in one part of the system will inevitably have ramifications for other parts. Increasing brain mass, for example, imposes additional energy and nutrient requirements, which in turn requires greater investment in foraging. Such energetic pressures can result in animals investing in riskier and more time consuming foraging, potentially exposing themselves to higher predation risk. These pressures further compress time budgets and their capacity to invest in other essential activities, including social bonding. Time is a major issue for animals, and especially for primates, and its significance should not be underestimated. Third, group-living imposes significant physiological costs on females, in particular, because of the impact that social stress has on menstrual system endocrinology, and hence infertility. In short, group size cannot simply be dismissed as a casual by-product of having a large brain—it is a central part of the story owing to feedback loops in the relationships between these variables.

Note that, for present purposes, we shall frame our discussion mainly in terms of brain size, rather than specifying particular brain regions. Even though primates' large brains are mainly the consequence of a dramatic increase in neocortex volume [20,21], most of the behavioural relationships we discuss correlate to some degree with almost any index of brain size, and we do not wish to be side-tracked into unnecessary debates as to whether some brain regions are more important than others. We note below that multiple measures of brain architecture support similar conclusions. The reasons why absolute brain size may the best measure to use as a proxy for cognitive capacity in primates have been discussed by other authors [17,18].

One final point is worth stressing: virtually all studies on this topic are based on correlational evidence. Evolutionary hypotheses have always suffered from the disadvantage that we cannot easily test causality in what are implicitly causal hypotheses. From time to time, experiments are conducted, but the substantive analyses based on these are always, of necessity, correlational (brain size correlates with task performance [18]). However, there are alternative approaches that can now be used, and we will argue that that these need to be given more attention.

## 2. Why and how large brains evolve

**Figure 1.**
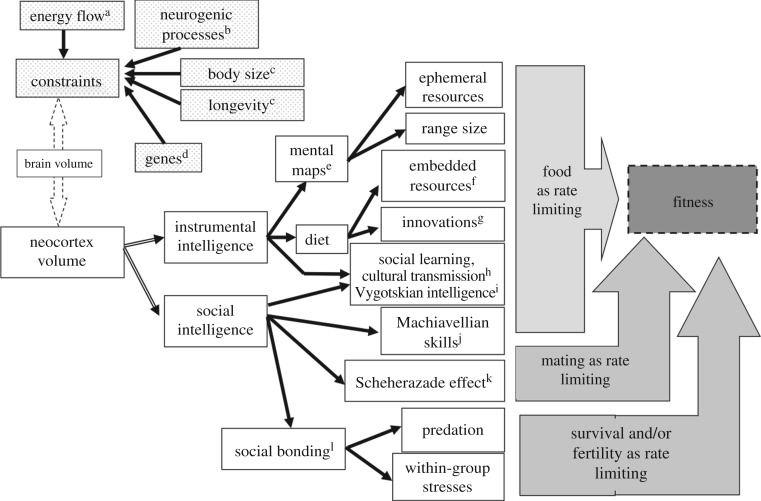
Alternative explanations for the evolution of large brains in primates. Explanations differ in whether their central claim is (*a*) about ontogenetic or energetic constraints, versus ecological or social processes, (*b*) whether they view food, mating or predation as the rate-limiting process for fitness, and (*c*) whether they view the fitness benefits from large brains as being direct (individual-level benefits) or indirect (arising out of social processes). Principal references: ^a^[22–24], ^b^[20,21], ^c^[22,25–27], ^d^[28–33], ^e^[25], ^f^[34], ^g^[17,18,35], ^h^[17,36,37], ^i^[38], ^j^[39], ^k^[40] and ^l^[5,6,8,41,42].

To provide a framework, we summarize the various hypotheses that have been proposed, and their functional implications, in figure 1. We structure this as a decision tree in which the options are narrowed down progressively from left to right.

In the top left corner, we list a number of factors that have often been proposed as *bona fide* hypotheses for brain evolution but which are, in reality, constraints on brain size rather than functional explanations. It is essential not to confuse mechanistic and functional explanations [2]. Evolutionary constraints typically operate via limited developmental opportunities (what possibilities are available given a current set of traits [43]). In terms of brain evolution, developmental constraints have focused on life-history traits [22,25–27] and neurogenic [20,21,44] explanations, but more recently genetic explanations (and especially those genes associated with accelerated evolution within the human lineage [28–33,45]) have joined this set. Evolutionary constraints can also involve the physiological costs to grow and maintain traits [23]. Brains are extremely expensive [46–48], and these constraints represent some of the costs that animals must be able absorb in order to evolve large brains *if* they have a compelling reason for doing so. Conventionally, these include metabolic rate [24,49], and energetic or dietary requirements [22,24,46,50,51]. Developing solutions to overcome these constraints are necessary, but not sufficient, explanations for the evolution of large brains.

All the remaining explanations in figure 1 are, in principle, functional explanations (i.e. they make claims about the selection forces that might have driven brain evolution). They divide naturally along two dimensions: first, by whether the animals solve their fitness-limiting problems individually (by trial-and-error learning or insight) or socially (the presence of several individuals is explicitly necessary for the solution to be effective), and then, secondarily, by whether the fitness-limiting factor is direct (the acquisition of food or mates) or indirect (e.g. ensuring group coordination so as to manage an exogenous threat to survival or fertility).

### (a) Instrumental hypotheses

These hypotheses focus mainly on the demands of food-finding and implicitly (but almost never explicitly) assume that foraging is the single most important constraint on an animal's fitness. In effect, this is the default position for ecologists. In early analyses, frugivory [25] was assumed to be cognitively more demanding than folivory, and it may well be: fruits are less predictable in time and space than leaves. However, phylogenetic comparative analyses find no relationship between the degree of dietary frugivory and brain size when controlling for social group size across mammals [6,34,52]—though the latter fact may be the crucial giveaway in that it may indicate that a change of diet is needed when large groups are involved because of the effect that group size has on energetics [19].

More importantly, perhaps, for smart foraging to have any traction as an explanation, it is necessary to show that primates do something different from non-primates—otherwise why would they need bigger brains than other mammals? For this reason, more recent studies have focused on foraging innovations, including the discovery and exploitation of novel foods [52] or novel means of accessing foods [34]. A number of analyses have shown that foraging innovations correlate with brain size in both birds and primates [17,53–55], and this relationship has in turn been related to species' abilities to survive in challenging habitats (birds [56–60], primates [61,62] and hominins [62–66]). The weakness of this claim is that most taxa do not in fact exhibit much smart foraging or technical innovativeness, despite variation in brain size across species.

The crucial fact is that, in primates, the relationship seems to be more of a phase transition: most species exhibit no innovations at all and a few exhibit a lot [17]. Given this, it would seem to be stretching a point to claim that what is in effect a dichotomy in innovativeness is responsible for a *quantitative* change in brain size across the entire order. An obvious alternative explanation might be that smart foraging is a by-product of acquiring a brain of a particular minimum size (i.e. breaking through a glass ceiling on brain evolution).

What evidence is there to suggest that ecological or technical decision-making has actual fitness consequences? Only one study has assessed this directly: Altmann [67,68] showed that female baboons who were better able to match an optimal diet (in terms of energy and protein intake) as *yearlings* survived longer, had longer reproductive careers and produced more offspring (whether indexed as total number of births or the number that survived to 12 months). Although the sample size is small (*n* = 6 females), the results are remarkably linear and convincing. Large brains certainly provide the *capacity* to engage in efficient trial-and-error problem-solving or insightful one-trial learning [69].

A more important issue concerns the assumption that food is, or by extension energy budgets are, the primary factor influencing an animal's fitness, either because all other extrinsic effects are trivial by comparison or because foraging is the only factor that an animal can actually control through its behaviour. In fact, for mammals generally, and primates in particular, predation is also a major consideration [70–72] and has a much greater effect on species' biogeographic distributions [19,73]—and this is widely so across mammals and birds generally.

Individually and collectively, instrumental hypotheses seem to fail as a general explanation because they do not explain why primates should need bigger brains than anyone else, why primate brains vary so much in size between species, why primates should have bonded social groups, why group size should correlate with brain size or why pairbonded species should need such big brains. On balance, then, foraging skills may be better explained as the solution to a constraint on the growth and maintenance of large brains when these are required for some other reason.

### (b) Social hypotheses

The social explanations place an explicit emphasis on sociality as the key issue associated with large brains (figure 1). There are at least five different versions: the original Machiavellian (or social) intelligence hypothesis (MIH) [39], the cultural intelligence hypothesis (CIH) [36,74–76], the Vygotskian intelligence hypothesis (VIH) [38], the Scheherazade hypothesis (SH) [40] and the social brain hypothesis (SBH) *sensu stricto* [5,41,42,77,78]. These five hypotheses differ in what they see as the central problem that big brains allow animals to solve and the mechanism(s) required to achieve this.

#### (i) Machiavellian intelligence hypothesis

The MIH was the earliest of these social explanations. As originally conceived by Humphrey [79] and later elaborated by Byrne & Whiten [39], MIH argues that primates live in inherently competitive social environments in which individuals compete with each other to steal food and/or mates. Tactical deception [80] became the defining criterion for this hypothesis, and its frequency does correlate with relative neocortex volume [81]. However, this explanation does not explain why primates live in social groups: if being exploited by group members is what happens when you live in a group, why would anyone choose to reside in bonded social groups [8]. Living in casual herds of indeterminate size should be sufficient to allow individuals to exploit each other in the way envisaged by the MIH. Species, like artiodactyls, that regularly form aggregations on rich pastures should exhibit Machiavellian behaviour and have large brains. But, in fact, they do not. Perhaps the real issue is that Machiavellian behaviour, being intrinsically competitive, is inherently socially destructive and thus likely to lead to the fragmentation of groups [81] unless some very significant counter-selection pressure exists to force animals to stay together despite their Machiavellian behaviour.

In sum, MIH might explain *how* primate sociality is different from that of all other mammals, but offers no explanation as to *why* this should be so. Indeed, one might argue that Machiavellian behaviour is more likely to be a consequence of living in large groups (that provide many individuals to exploit) rather than its cause. MIH seems to fail because it is not of itself intrinsically social, and does not explain why group size (and hence brain size) should vary across primates (or why groups are bonded and continue to stay together).

#### (ii) Cultural intelligence hypothesis

CIH is a more explicitly social version of the smart foraging hypothesis. The central claim is that large brains enable the social transmission of behaviour (or, more generally, information), principally by imitation or mimicry [17,35–37,53,76]. Inevitably, CIH faces the same problems encountered by instrumental foraging explanations: social groups (or networks) are necessary for the propagation of information, but are not sufficient as an explanation for the evolution of group-living. Since group-living is intensely costly for animals, and especially so for primates (see below), we must ask why animals are prepared to pay these costs as well as the energetic costs of large brains simply in order to exchange information.

The crucial issue is whether animals that are better at absorbing social information have higher fitness. Altmann's [67,68] study of optimal foraging in yearling female baboons is, once again, the only relevant evidence. The fact that individual foraging skills as *yearlings* (the age at which weaning occurs in baboons) predict lifetime fitness would seem to suggest that, if anything, foraging skills are learned *before* being weaned and are less likely to be influenced by cultural experiences during either the main period of socialization (weaning to puberty) or in adulthood when cultural transmission should be at its most important.

Aside from this, CIH often appears to refer to rather a narrow conception of complex learned behaviours (including technical behaviours like tool use). Moreover, the fact that both technical competence and cultural transmission are step-like rather than continuous in their distribution within primates [17] seems to suggest that these explanations may be relevant for a small handful of species (great apes, humans?), but not for all. Muthukrishna & Henrich [76] certainly provide compelling evidence for the importance of culture and cultural transmission as an explanation for the fact that human communities and achievements are many orders of magnitude greater than those of other primates. However, the evidence they adduce for the claim that population size affects innovation rates all derives from modern societies (i.e. post-Neolithic and contemporary societies). By contrast, the evidence that brains evolved for innovativeness before the Neolithic is not impressive: tool manufacture has very long periods of stasis in the archaeological record and does not correlate well with the evolution of human cranial capacity [82,83]. That said, cultural icons and their transmission do play a singularly important role in social bonding at both the dyadic and the communal levels in humans ([84]; see also [85–90]), which would perhaps make this a version of the SBH (see below) where culture becomes part of the bonding process.

CIH has been extended to include cooperative breeding [91,92] on the grounds that cooperative breeding imposes coordination and investment challenges, as well as the potential to extend development in a way that might facilitate social learning. The anatomical evidence to support this claim is, however, mixed. Even though obligately monogamous species (e.g. indrids, cebids and hylobatids) do have larger brains than we might expect for their group size [93,94], the males make little if any direct contribution to rearing in these species; by contrast, genuinely cooperative breeders like callitrichids (and maybe humans), where the male plays an active role in rearing, actually have smaller than expected brain sizes [94,95]. By the same token, monogamous ungulates have larger brains than polygamous/promiscuous ungulates [58,96], yet in no species does the male provide any paternal care. This perhaps suggests that the cognitive demand may have more to do with pairbonding (i.e. behavioural coordination) than cooperative rearing as such [7]. If the issue really is pair coordination, then the proposal is simply a small-scale version of the SBH (see below), where precisely this claim has previously been made for mammals and birds, in general [7].

There are two further considerations in respect of CIH that we should note. First, although the occurrence of cultural transmission does correlate with social group size in primates, no evidence has yet been offered to suggest that the efficiency of innovation or transmission actually increases with the number of models available. Indeed, if anything, the converse may be true: the natural structuring of primate social groups actually *slows down* the rate at which innovations spread through a group [97,98]. Second, the fact that animals ‘infect’ each other with novel foraging strategies, technical know-how or cultural rules does not, of itself, tell us anything about why (bonded) social groups exist. This is not to say that the flow of information through networks is not important and does not have functional (i.e. fitness) consequences [37,99]; an equally plausible claim is that the use of socially available information is an exaptation, or secondary benefit, of group living.

In sum, as an explanation, CIH has three issues: it seems to apply only to a very limited number of species; it cannot explain the variation in nonhuman primate brain size (especially given that the kinds of phenomena likely to be transmitted culturally, such as innovations, seem to have a stepwise rather than continuous distribution in primates [17]) or why primates should have bonded social groups. That it might be a by-product benefit (or evolutionary window of opportunity) to one of the other hypotheses is, however, a distinct possibility.

#### (iii) Vygotskian intelligence hypothesis

VIH was proposed by Moll & Tomasello [38]. They argued that all non-human primate societies are essentially competitive; hence, the MIH (as originally proposed by Humphrey [79]) provides a sufficient explanation for the evolution of large brains in nonhuman primates. By contrast, they claim, human societies are intrinsically cooperative and for this a new kind of intelligence (Vygotskian intelligence) was needed. Vygotsky [100] developed a theory of culture during the 1930s that emphasized the social aspects of intelligence, in particular collaboration, communication and teaching. Moll & Tomasello [38] argued that this form of intelligence is unique to humans and marks a phase shift both in how social life is organized and in the kinds of cognitive demands placed on the brain, thereby explaining the fact that humans have brains that are significantly larger than those of all other primates.

Appealing as this suggestion is, it falls foul of three problems. First, Moll & Tomasello [38] misunderstand the nature of primate sociality. Contrary to what they assume, all (anthropoid) primate societies are in fact based on cooperation: primate groups are cooperative solutions to the central problems of survival, in particular predation risk. Second, as originally stated, VIH argues a special case for a single taxon: special pleading should always be an explanation of last resort. Third, as with so many other hypotheses, it fails to explain why group size varies across primates, or why it correlates so robustly with brain size. One could, of course, argue that the Vygotskian model applies to all primates. But since it appears to be a categorical phenomenon (you have Vygotskian intelligence or you do not), it would then be difficult to explain the quantitative differences in brain size across primates. Nonetheless, VIH may well be a plausible explanation for the mechanism(s) needed to ramp conventional primate social intelligence up onto the higher plane needed to manage the substantially larger scale of human societies.

#### (iv) Scheherazade hypothesis

SH argues that human's very large brains arose as a consequence of sexual selection in the context of managing mate fidelity [40]. However, we can exclude the possibility that sexual selection for mate quality has driven brain evolution in primates as a whole: brain size does not correlate with at least two well-established indices of sexual selection, namely relative testis size and the degree of female sexual promiscuity [93]. Nonetheless, it might possibly offer an explanation for the evolution of the large brains characteristic of monogamous mammals and birds [7,101,102] if we can argue that the issue is selection for mate *bonding* (the processes that underpin behavioural coordination) rather than mate *selection* (for genetic quality) [9]. This would explain why, in birds, lifelong pairbonders have significantly larger brains than annual pairbonders who find a new mate each year [7]. The bottom line is that if SH's scope is simply as an explanation for the very large brains of modern humans, its interest may be limited; but, if it offers a more general mechanism for pairbonding, then it may be a version of the SBH (see below). In this respect, however, it potentially adds an important way in which the SBH can be generalized to a taxonomically wider range of species beyond the primates [7].

#### (v) Social brain hypothesis

SBH has been used (or misused) to refer to many different, often rather vague, hypotheses. We ignore most of these and focus on SBH *sensu stricto*, defined as originally specified in terms of the need to create functional, cohesive, bonded social groups as a means of solving an ecological problem [41]. SBH is an explicitly two-step process: an ecological problem is solved socially (i.e. as a cooperative process) and a big brain is needed to allow the requisite level of sociality to do this. The group created by big brains is not an end in itself (a mistake made by many attempts to compare between alternative hypotheses: e.g. [18,103]), but rather the means to an ecological end. It is important not to mistake this view for group selection: it does not involve the differential survival or extinction of groups. Rather, it is a group-level or group-augmentation explanation (*sensu* [104]), such that individuals living in larger social units have higher fitness than those living in smaller ones.

It is worth rehearsing briefly the weight of evidence for the SBH, and how remarkably robust the relationship actually is. In primates, social group size correlates with a wide range of brain indices, including absolute and relative cranial volume [105], brain volume [7,106], neocortex volume [5,10,42,107], non-striate neocortex volume [108] and frontal lobe volume [109] (with and without phylogenetic correction in all these cases and with roughly similar goodness of fit), in several different datasets, in many cases while simultaneously controlling for a variety of potential ecological confounds [7,58,96,106]. The correlation is significantly improved if group size is indexed as female cohort size [110], suggesting that it may have been female grouping patterns that have driven brain evolution. It is improved still further by noting that the data actually form a series of grades [109,111]. (By grades, we refer to the fact that the regression equations for two subsets of data have the same slope but significantly different intercepts: see [111].) Many studies that compare social group size against other cognitive or behavioural measures as predictors of brain size (e.g. [34,103]) treat the SBH relationship as a single unitary equation relating group size to brain size. Using a single generic regression, equation yields a significantly poorer fit, as grades inevitably result in regression slopes being pulled down [112]. It is essential to match species to the correct grade when undertaking such tests if egregious errors are to be avoided.

In addition, and more impressively, there is now considerable neuroimaging evidence for both humans [113–118] and macaques [119] that *individual* differences in social network size (variously indexed as sociability, core social network size, number of Facebook friends and living group size) correlate with the absolute volume of core regions in the frontal and temporal lobes of the brain. Thus, the social brain relationship applies not just between species but also, within species, between individuals (as might be expected of any trait subject to natural selection). These results also narrow down the focus of the correlation and identify those brain regions associated with social skills (in particular, in the frontal lobes) as being critical to the relationship with group size.

For reasons that are not entirely clear, SBH is frequently perceived as being just about group size. In fact, right from the outset SBH explicitly claimed that group size is an emergent property of the animals' abilities to maintain and coordinate social relationships [40]. This is evident from the fact that neocortex volume correlates with a number of behavioural indices of social complexity in primates, including the size of grooming cliques [13,120], the use of coalitions [6], the use of sophisticated social strategies [121] and the frequency of tactical deception [81]. Importantly, there is direct evidence that individual differences in social skills have real implications for fitness [121–125]. In baboons, female longevity *and* fecundity correlate with the number of social partners that females have [122–124], and females with more grooming partners are better able to cope with stressful events (as indexed by cortisol titres [126]). There is now extensive evidence that social network size and quality are among the most important factors influencing health, wellbeing and even longevity in humans (for a recent summary, see [127]).

Although SBH was originally developed as an explanation for the evolution of primate brain size, a number of studies have explored its implications for other taxa. However, Pérez-Barbería *et al*. [10] and Shultz & Dunbar [7] demonstrated that primates differ radically from carnivores and ungulates in their pattern of brain size evolution. Only anthropoid primates exhibit a quantitative relationship between group size and brain size; in all other mammals and birds, SBH is instantiated as a categorical difference between pairbonded species with big brains and polygamous species with small brains [7,8,96,102,128]. Moreover, Shultz & Dunbar [8] showed that the historical rate of encephalization over geological time within different mammalian sub-orders correlates with the proportion of living genera that have bonded social systems (defined as either pairbonded or groups in which individuals have a limited number of preferred social partners, resulting in highly structured networks). Some taxa, such as the felids (almost all of whose living species are solitary), show little evidence of encephalization across their entire geological history, whereas taxa like the canids and anthropoid primates that have a high frequency of bonded groups show an accelerating change in encephalization. Given that it has repeatedly been shown that the quantitative relationship between brain size and group size does not apply outside the anthropoid primates [7,10], it is puzzling that social group size has so often been considered an appropriate index to correlate with brain size across widely divergent vertebrate taxa (for recent examples, see [18,103,129]).

Sociality may also have been instrumental in selecting for large brains in some fish families (e.g. the cichlids: [130]) and among the social insects ([131,132], but see [133]). Again, group size is not the issue in either case, but rather social skills. Bee and wasp species in which the queens are social (several queens share a nest) have larger mushroom bodies (the part of the brain that handles social behaviour) than species that nest solitarily, and within these social species queens have larger mushroom bodies than workers. Gonzalez-Voyer *et al*.'s [130] study of cichlids is unique in that it used a selection experimental paradigm to show that, at least among females, selection for social skills resulted in changes in brain size.

As a final aside, we note that, although it has been claimed that CIH represents a novel approach, because it highlights the role of social learning [39], in actual fact social learning and the practice of social skills was identified at a very early stage as being a crucial component of SBH [4,41,134,135]. The skills required to maintain social coordination and cohesion are complex and have to be learned. There is abundant evidence from both developmental psychology [136] and neuroimaging [137] that, in humans, this actually takes a very long time (possibly as long as two decades). The fact that, in primates, neocortex volume is best predicted by the length of the socialization period (weaning to first reproduction), and not by the period of parental investment (gestation plus lactation) that conventionally explains total brain size [134], reinforces this point. It is not enough to have a big computer; the computer needs software, and the software is acquired by learning, imitation and practice. Herein may lie the substantive importance of cultural transmission and CIH.

## 3. Towards an integrated model

To avoid the apparent bind caused by the need to differentiate between alternative explanations, some authors have suggested that the brain evolved to support several or all of the functions reviewed in §2, and hence that the distinction between ecological and social explanations is a false dichotomy (e.g. [37,138]). At a cognitive level, this is almost certainly true: the same cognitive processes very likely do support all these different functions, since processes like causal reasoning, analogical reasoning, one trial learning, the comparison of alternative outcomes and the ability to inhibit prepotent responses underlie all forms of primate decision making, whether social or instrumental [139]. More importantly, these are unique anthropoid competences dependent on Brodman area 10 in the frontal pole, a brain region that exists *only* in anthropoid primates [69]. Indeed, these executive function competences correlate with neocortex volume across primates [106]. It may not, however, necessarily be so where functional explanations are concerned. Here, an important distinction will usually need to be drawn between the function that led to the original evolution of a particular trait, and the function(s) that have subsequently been responsible for coopting the trait for other biological purposes.

One of the problems in this respect is that, because social skills are more nebulous to define and their benefits typically accrue only on the scale of a lifetime, foraging tasks have been much easier to work with in both the laboratory [106,140,141] and the field [142]. This makes it difficult to design experiments that genuinely distinguish between technical (instrumental) and social hypotheses. Although limited to apes, the one serious attempt to test between competences on technical and social competences directly [74] reveals a remarkably linear relationship between prefrontal cortex volume and performance on a genuinely social task in three ape species (orangutans, chimpanzees and humans), whereas competences on non-social instrumental tasks exhibit, at best, a stepwise relationship with brain size. These differences in social skills also correlate monotonically with group size, whereas those for instrumental skills do not. More data of this kind are clearly needed on other primate species, but such experiments are not easy to design.

A further problem in testing between alternative hypotheses is that almost all comparative analyses are, inevitably, correlational, making it impossible to test causal hypotheses. This problem has been somewhat ameliorated recently by the development of new phylogenetic statistical methods that allow causal hypotheses to be tested by examining the sequential order in which correlated variables appear in the phylogenetic tree (e.g. [143,144]). Broadly speaking, this works fine so long as cause and effect are separated by time intervals long enough to be picked up by the phylogenetic timescale. In many cases, however, the coevolutionary process is so tightly locked that there is insufficient temporal precision to detect a difference [10].

Even so, when doing so, it is important that both the hypotheses *and* their behavioural indices are covalent (i.e. of equal logical standing). This is especially true if we use multiple regression to compare the influence of two alternative behavioural indices. Recent examples where this desideratum has not been observed include: Bailey & Geary [63], who used multiple regression to test between environmental instability (an index of energy challenge: an environmental driver) and social competition (with fossil abundance used as a proxy by population density: a consequence) in hominin brain evolution; Charvet & Finlay [44], who tested between body weight (an index of energy flow: a constraint) and social group size (a function); MacLean *et al*. [18], who tested between behavioural inhibition (a cognitive variable, and hence a mechanism) and social group size (a function) in mammals as a whole; and Benson-Amram *et al*. [103], who tested between problem-solving ability (a cognitive measure) and social group size (a functional index) in hyaenids (notwithstanding the fact that, in the latter two cases, SBH does not apply as a quantitative relationship to mammals as a whole). All conclude that some aspect of environmental conditions is the main (or only) driver of brain evolution. In one sense, that goes without saying, since even the SBH is an ecological hypothesis (groups exist to solve an ecological problem: the issue is do the animals solve the ecological problem socially or by individual skills). Confusing one kind of explanation with another is a type of logical error known as a category mistake.

**Figure 2.**
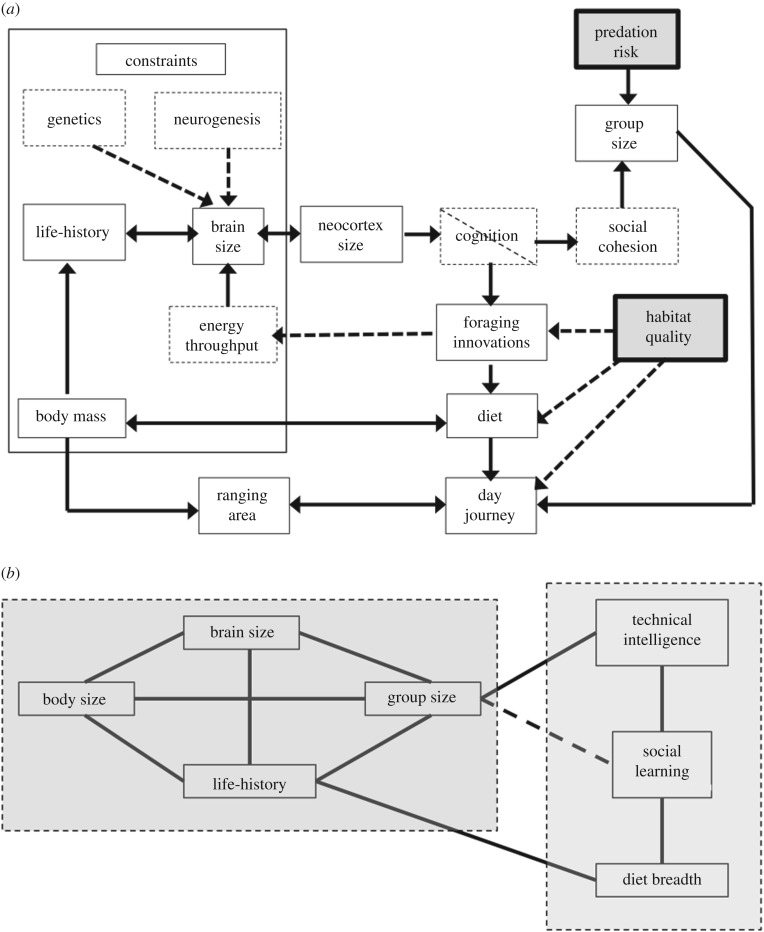
(*a*) Path model of Dunbar & Shultz [6] defining the relationships between key variables in primate social, ecological and brain evolution. Solid lines indicate statistically confirmed relationships based on the phylogenetically controlled path analysis given by Dunbar & Shultz [6]; dotted lines indicate additional relationships not included in the original path analysis but for which there is confirmatory statistical evidence. Boxes with dashed outlines indicate variables not included in the original path analysis of [6]. Shaded boxes indicate major environmental drivers. (*b*) Path model of Navarette *et al*. [52]. Lines indicate phylogenetically controlled statistically significant relationships. Technical intelligence here refers to a combination of foraging innovations and extractive foraging. In some (but not all) models, social learning correlates with social group size (dashed line). Dashed boxes enclose variables that covary together. The graph is redrawn to a similar orientation to that in (*a*).

An alternative approach is needed. A particularly suitable one is path analysis (or structural equation modelling), since it is explicitly designed to handle conceptual nesting of this kind. Path analysis uses the partial standardized slopes from multiple regression equations to test between alternative causal relationships that link a set of variables. One attempt to do this [6] is illustrated in figure 2*a*. This suggests that predation risk is the main driver for group size, which in turn selects for the cognition needed to support social groups, which in turn selects for the neocortex size needed to support this kind of cognition. Since large neocortices can only be supported by large brains, this has energetic consequences that need to be met, with enhanced ecological problem-solving abilities as a consequence. Most of the life-history and ecological variables form a set of energy-related constraints or costs that have to be resolved to achieve any increase in brain volume.

Recently, Navarette *et al*. [52] used phylogenetically informed causal graphs to explore the functional relationships between social group size, brain size (they did not consider neocortex volume), other life-history variables and a number of relevant foraging variables (including technical feeding innovations, social learning and diet breadth; figure 2*b*). Here, innovations, social learning and diet breadth turn out to form one functional module, while social group size, brain size, body size and life-history form another. Although social learning does not correlate independently with group size in all models, in one model it did, perhaps suggesting, as implied by CIH, that the number of models available may be an important factor influencing the extent to which social learning can manifest itself *or*, as implied by the suggestion that nutrient throughput is a constraint (§2a), that smart foraging is a requirement for the evolution of large brains when the costs of living in large groups put pressure on nutrient throughput.

Although taking different approaches and using different variables, these two path analyses come to essentially the same conclusion: brain evolution is driven largely by social factors, with instrumental skills being a beneficial spin-off (probably because they exploit the same cognitive mechanisms) which is nonetheless essential because they solve the inevitable energetic bind that large brains necessarily entail. Increasing body mass to support a bigger brain [50] may bring with it well known savings of scale in terms of basal metabolic rate that allow surplus energy to be diverted to fuelling brain growth and maintenance [24,145], but this does not obviate the fact that bigger bodies consume absolutely more energy.

In sum, the set of variables that have been correlated with brain size are part of a single, integrated biological system whose components are all essential, but which play importantly different roles in the explanatory story. The feedback loops in this model explain why all variables correlate as well as they do.

## 4. What makes primate sociality so different?

We identified primate sociality as a key element in this story: so what is it about primate sociality that is so cognitively demanding as to require a large brain? We address this question in more detail elsewhere, but for present purposes we highlight two key features of primate sociality that we see as being crucially different from most other mammals and birds, and which are likely to be cognitively very demanding.

One is the fact that primate social groups exist to provide a passive defence against predators [70,146–150]; in primates, in particular, group size is adjusted to the level of experienced predation risk [73]. Although such groups do not incur the conventional costs of public goods (animals do not pay a contribution to benefit, and hence cannot freeride), they do require individuals to coordinate their activity schedules if they want to be part of the group. Failure to do so will result in the group very quickly disintegrating as individual animals drift apart. This is a much more serious problem than many appreciate. In ungulates, herds rapidly disintegrate because the activity schedules of different individuals do not coincide [151–153]. Sex differences in energy demand, and hence required feeding time, owing to differential reproductive costs or differences in body mass are a particular problem: when some individuals need to continue feeding or move to new feeding sites but others want to rest, someone has to give way if the group is to stay together. The ability to inhibit prepotent responses (a correlate of neocortex volume, and Brodman area 10 in particular [69,106]) is likely to be crucial in this context. These costs are not trivial, because very few species have significant quantities of free (i.e. uncommitted) time [19].

We have highlighted the fact that energy demand is limiting for brain evolution. However, it is also limiting for another important reason, namely the fact that both foraging and social bonding are time consuming: though not widely appreciated, large-bodied mammals (and diurnal primates, in particular) may be more limited by time than by energy [19]. As a result, time is a major determinant of anthropoid biogeography [19,71,73,154–156]. Owing to the need to devote time to leaf fermentation, folivorous *Colobus* monkeys, for example, are unable to live in large groups because they do not have sufficient time to devote to social grooming at the level required to bond larger groups; modelling shows that if they switched to a more frugivorous diet, as their sister-genus *Piliocolobus* has done, they would free off sufficient time to live in groups as large as those found in *Piliocolobus* [154].

The second issue is a general problem facing all social animals: living in groups creates intense social stresses that arise in part through ecological competition and in part simply through the consequences of crowding in limited space. These create strong dispersive forces that inexorably precipitate group fission if they are not defused. Some of these are owing to the effects of energy bottlenecks (low ranking animals are excluded from the best food sources); others arise as a consequence of the effects that even low levels of harassment have on female menstrual endocrinology [157,158] and consequential loss of fertility [159–162]. These fertility costs are not widely appreciated, but they have very significant consequences for females' fitnesses. If they are not mitigated, females risk being functionally infertile.

Normally, these stresses are defused by animals leaving the group (the solution widely adopted in fission–fusion forms of sociality, even among primates [15]). However, most primates do not have fission–fusion sociality. Instead, anthropoid primates solve these problems through grooming-based coalitions that protect their members against harassment by other individuals in the group [163]. These coalitions provide both active defence when threatened by others (e.g. gelada [164]) and a form of passive deterrence (monkeys are less likely to attack individuals who have powerful allies, even when those allies are not in view [165]). Being able to keep absent individuals in mind at the same time as physically present individuals is cognitively very demanding. This is equivalent to being able to compare the outcome of two different behavioural strategies, and depends on both being able to inhibit prepotent action (holding back from attacking an individual one might be able to displace easily on a one-to-one basis) as well as modelling a virtual reality.

The prevalence of this relatively unusual form of bonded relationship [9,12] has the important consequence of creating the distinctive layered structure of primate social groups [13–16]. The result is a multi-level form of social organization similar to that found in humans [16,166–169]. In effect, these species are able to maintain two qualitatively distinct kinds of relationship simultaneously: intimate relationships with principal grooming partners (allies) and weaker ones with other group members with whom they do not often interact directly. This resembles the two-tier structure of human social relationships, where parallel distinctions are drawn between weak and strong ‘ties’ [170,171] and, orthogonally, between family and friends [172,173]. At least some Old World monkeys are able to factor two separate social dimensions into their calculations about others' status (e.g. baboons [174]).

The need to be able to manage relationships of two different types (those based on direct interaction, and those inferred from third-party interactions) is cognitively demanding, and in humans forms the basis of the ability known as mentalizing (or theory of mind) and the ability to model the social world in a virtual mental environment. Not only do individuals' mentalizing abilities correlate with the size of their social networks in humans [114,175], but they also correlate with the volume of core regions in the brain, especially in the prefrontal cortex and the temporal lobes [113,115]. While formal theory of mind is probably an exclusively human (but possibly also great ape [176–178]) competence, the capacity to handle alternative causal sequences (i.e. propositionally structured descriptions of the world) is a uniquely anthropoid ability [69].

That these social cognitive abilities may form a scaled continuum across anthropoids is implied by the fact that comparative neuroimaging studies indicate that the human and macaque neocortex is organized in the same way for social functions [179]. Indeed, tractography studies have recently shown that humans and macaques have the same white matter tracts connecting the ventral prefrontal cortex with the temporal lobes that, in humans, are associated with the theory of mind (or mentalizing) network [180]. This brings us back full circle. This kind of cognitive complexity is not an all-or-none phenomenon like formal theory of mind, but a quantitatively varying one that is directly related to group size [109]. As the demand for this increases with group size, it necessitates a proportionally bigger brain to support it, and this adds significantly to the energetic burden that the animal must find additional foraging time to offset.

## 5. Conclusion

Table 1 identifies which of the six criteria listed in §1 are satisfied by each of the hypotheses. Only SBH satisfies all the criteria. Because SBH *sensu stricto* is about the quality of relationships (and their functionality as coalitions) and not simply about group size, this hypothesis naturally also explains the evolution of pairbonding in non-primates, thus providing a unitary explanation for brain evolution across all mammals and birds. At the same time, it is clear that the other components (or hypotheses) play significant structural roles.

**Table 1.** Comparison of the main hypotheses for brain evolution against the five key explanatory criteria.

	hypothesis can explain^a^					
criteria	instrumental	MIH	CIH	VIH	SH	SBH
(i) primates have larger brains that other animals	X	√	X	(√)	(√)	√
(ii) quantitative variation in primate brain size	√	(√)	X	X	X	√
(iii) brain size correlates with group size in primates	X	X	X	X	X	√
(iv) primate sociality is complex (bonded)	X	X	X	X	(√)	√
(v) pairbonded non-primates have large brains	X	X	X	X	√	√
(vi) some primates are more innovative technically	√	X	√	X	X	√

^a^√, the hypothesis provides an explanation for the phenomenon indicated; X, hypothesis is unable to account for the phenomenon. Parentheses indicate cases where the evidence is arguable.

This proposal has several advantages. First, it integrates a wide range of data of very different kinds into a single explanatory framework, and provides a reason why there should be evidence to support all the hypotheses that have been proposed. Second, it provides an explicit explanation for the relationship between social group size and brain size, and why primate brain sizes should vary quantitatively across species. Third, it provides an account of the kinds of behaviour and cognition needed to maintain bonded social groups, reasons why these should be cognitively more demanding than more conventional instrumental skills and how these relate to the underlying neurobiology. Fourth, it offers reasons why social learning (and hence cultural transmission) should be important. Fifth, and more importantly perhaps, it provides an explanation that spans the full range of birds and mammals, arguing only that pressures to evolve bonded relationships reflect the particular circumstances and evolutionary histories of individual taxa. Individual species may well be subject to other selection pressures that add or subtract from this overall effect, but there is no need to plead special cases.

Finally, perhaps, we can answer the question with which we started: why are there so many different explanations for primate brain evolution? Put simply, too many analyses focus on correlational evidence while advocating a single explanation to the exclusion of all others; as a result, they rarely make serious attempts to integrate the various explanations (for all of which there is convincing evidence) into a single unitary explanatory framework. We hope that we have gone some way towards achieving that objective.
